# Metal Tolerance Protein Encoding Gene Family in *Fagopyrum tartaricum*: Genome-Wide Identification, Characterization and Expression under Multiple Metal Stresses

**DOI:** 10.3390/plants11070850

**Published:** 2022-03-23

**Authors:** Zhiqiang Li, Chenglong Wang, Kaiyi Wang, Jiayu Zhao, Jirong Shao, Hui Chen, Meiliang Zhou, Xuemei Zhu

**Affiliations:** 1 College of Environmental Sciences, Sichuan Agricultural University, Chengdu 611130, China; 2019107001@stu.sicau.edu.cn (Z.L.); 2019207005@stu.sicau.edu.cn (K.W.); 2020207002@stu.sicau.edu.cn (J.Z.); 2 School of Landscape Architecture, Beijing Forestry University, Beijing 100083, China; wangchenglong@bfus.com.cn; 3 Post-Doctoral Research Station, Beijing Forestry University Forest Science Co., Ltd., Beijing 100083, China; 4 College of Life Science, Sichuan Agricultural University, Ya’an 625014, China; shaojr007@163.com (J.S.); chenhui@sicau.edu.cn (H.C.); 5 Institute of Crop Sciences, Chinese Academy of Agricultural Sciences, Beijing 100081, China

**Keywords:** *Fagopyrum tataricum*, gene expression, metal stress, metal tolerance protein

## Abstract

Metal tolerance proteins (MTP) as divalent cation transporters are essential for plant metal tolerance and homeostasis. However, the characterization and the definitive phylogeny of the MTP gene family in *Fagopyrum tartaricum*, and their roles in response to metal stress are still unknown. In the present study, MTP genes in *Fagopyrum tartaricum* were identified, and their phylogenetic relationships, structural characteristics, physicochemical parameters, as well as expression profiles under five metal stresses including Fe, Mn, Cu, Zn, and Cd were also investigated. Phylogenetic relationship analysis showed that 12 *Fagopyrum tartaricum* MTP genes were classified into three major clusters and seven groups. All *FtMTP*s had typical structural features of the MTP gene family and were predicted to be located in the cell vacuole. The upstream region of *FtMTP*s contained abundant cis-acting elements, implying their functions in development progress and stress response. Tissue-specific expression analysis results indicated the regulation of *FtMTP*s in the growth and development of *Fagopyrum tataricum*. Besides, the expression of most *FtMTP* genes could be induced by multiple metals and showed different expression patterns under at least two metal stresses. These findings provide useful information for the research of the metal tolerance mechanism and genetic improvement of *Fagopyrum tataricum*.

## 1. Introduction

Trace elements, including metallic elements such as iron (Fe), manganese (Mn), copper (Cu), and zinc (Zn), are important to maintain normal growth and development in plants [1,2]. However, the growth and production of plants could be inhibited if they absorb excessive amounts of these trace elements [3]. In addition, some toxic heavy metal ions, including lead (Pb) and cadmium (Cd), could also be absorbed by plants because these metal ions have similar chemical structures with trace elements [4]. Recently, due to the rapid development of global industrialization and urbanization, the average content of metallic elements, including heavy metal elements in soil, have gradually increased, causing heavy metal stresses and affecting crop production [5,6]. Precise homeostasis of metallic elements is essential for plant growth and development [7,8,9,10].

The cation diffusion facilitator (CDF) family, which has been widely found in all six kingdoms of living things, plays a vital role in metal tolerance and homeostasis of organisms [11,12]. There are three common features in the CDF family members: an N-terminal signature sequence, a cation efflux domain, and approximately six predicted transmembrane regions [13]. Meanwhile, the CDF family members were clustered into three categories, called Zn-CDF, Zn/Fe-CD,F and Mn-CDF, which had different substrate specificities [14]. In plants, CDF family members are considered metal tolerance proteins (MTP). There are 12 and 10 members in *Arabidopsis thaliana* and *Oryza sativa*, respectively [15]. These MTP proteins have been found to be involved in transporting various metallic elements, including Fe, Mn, Cu, Zn, Ni, Co and Cd [16]. For instance, AtMTP1 and AtMTP3, two members of Zn-CDF, play essential roles in Zn and Co tolerance by transporting Zn^2+^ and Co^2+^ to the vacuole [17,18,19,20], while two more genes (AtMTP5 and AtMTP12) of Zn-CDF forms a functional complex during Zn^2+^ transportation into Golgi [21]. MTP8 is the most thoroughly investigated Mn-CDF family members, which contributes to Mn detoxification in *A. thaliana* and *O. sativa* by sequestering Mn in vacuoles [22,23,24]. Up to now, many MTP gene family members have already been found in non-model plants species, including *Sorghum bicolor* [25], *Vitis vinifera* [26], and *Camellia sinensis* [27], etc. However, the functional assignment of these MTP proteins identified recently are still unknown.

Tartary buckwheat (*Fagopyrum tataricum Gaertn*) belongs to the genus Fagopyrum in the family *Polygonaceae*. As a traditional and irreplaceable crop [6], it is widely cultivated in many countries, including China, Japan, South Korea, India, and Russia [28]. Due to its vital nutritional and medicinal values, it has been processed into a variety of foods, including tea, noodles, and shakima, which are popular all over the world [29]. However, it was reported that Tartary buckwheat had the ability to uptake toxic metals (Pb, Cd, and Hg) from the growth medium and accumulate them in the edible parts [30,31,32,33], and our previous research also found excessively high levels of some heavy metals in Tartary buckwheat tea [34]. These results suggest that heavy metal stress is one of the non-negligible factors restricting Tartary buckwheat production and food safety. Although a few previous studies have shown that Tartary buckwheat seedlings increase the activities of some antioxidant enzymes in response to oxidative stress caused by heavy metals [33] and promote the compartmentalization of heavy metals in vacuoles to reduce heavy metal concentrations in protoplasts [31], the molecular mechanism of heavy metal tolerance and detoxification of Tartary buckwheat is still largely unknown.

Thus, the purposes of this study are to (1) identify MTP gene family members from the genome of Tartary buckwheat; (2) systematically analyze sequences and structural characteristics of Tartary buckwheat MTP gene family members; (3) perform expression analysis of Tartary buckwheat MTP genes in response to various metal stresses. Results of this study will not only provide useful information for subsequent studies on the mechanism involved in heavy metal tolerance of Tartary buckwheat but also contribute to the genetic improvement of Tartary buckwheat.

## 2. Results

### 2.1. Identification and Physicochemical Parameters of MTPs in the Tartary Buckwheat Genome

According to the similarities with *Arabidopsis MTP* genes and MTP gene family features, 12 *Fagopyrum tataricum MTP* (*FtMTP*) genes were successfully identified from the Tartary buckwheat genome. Subsequently, based on their homology with the corresponding *Arabidopsis MTP* genes, these *FtMTP* genes were named *FtMTP1*, *FtMTP3.1*, *FtMTP3.2*, *FtMTP5*, *FtMTP6*, *FtMTP7.1*, *FtMTP7.2*, *FtMTP8.1*, *FtMTP8.2*, *FtMTP9*, *FtMTP11*, and *FtMTP12*, respectively. Moreover, sequence analysis was performed to investigate the characteristics of the *FtMTP* gene family. As shown in Table 1, the peptide length of FtMTP proteins ranged from 230 to 722 amino acids, with FtMTP12 and FtMTP5 having the largest and smallest length, respectively. The isoelectric point of FtMTP7.1 was predicted to be 9.23, whereas the isoelectric points of the remaining 11 FtMTP proteins were below 7.00. Besides, of the 12 FtMTP proteins, the predicted molecular weight of FtMTP proteins ranged from 25.41 to 80.51 KDa, and the numbers of predicted transmembrane helices ranged from 1 (FtMTP6) to 10 (FtMTP12). The grand average of hydropathicity (GRAVY) was an index reflecting the hydrophobicity of a protein; the GRAVY value greater than zero indicated that the protein was predicted to be hydrophobic protein, while less than zero indicated the protein was predicted to be hydrophilic protein. Regarding the GRAVY values of the FtMTP proteins, four were hydrophobic, and eight were hydrophilic. In addition, the predicted subcellular localization results showed that all FtMTP proteins were located on the vacuolar membrane, and FtMTP9 was also located on the cell membrane.

### 2.2. Phylogenetic Tree of 67 MTP Proteins from Six Species

**Figure 1:**
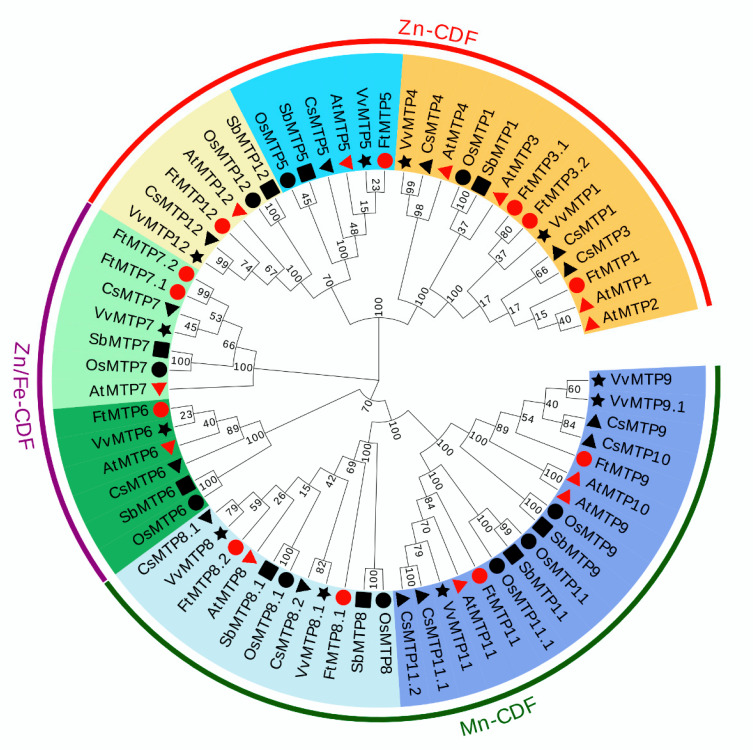
A phylogenetic tree based on 67 MTP proteins: 12 *Arabidopsis* (marked by red triangle), 10 rice (black circle), 11 grape (black star), 9 sorghum (black square), 13 tea plant (black triangle), and 12 Tartary buckwheat (red circle). The phylogenetic tree was constructed using the neighbor-joining (NJ) method and MEGA 7.0 software at 1000 replications bootstrap. The diagrams were compiled, labeled, color-coded, and visualized using EvolView v3.

In order to investigate the definitive phylogenetic relationship among MTP gene family members, the phylogenetic tree constructed of 67 MTP proteins from six species, Tartary buckwheat (12), *Arabidopsis* (12), Rice (10), Sorghum (9), Tea plant (13), and Grape (11) was investigated. According to their similarities at protein level, these 67 MTP proteins were clustered into three major clusters, including Zn-CDFs, Zn/Fe-CDFs and Mn-CDFs, and seven groups, namely group 1, 5, 6, 7, 8, 9 and 12 (Figure 1). However, the numbers of MTP protein in each group, as well as the numbers of group in each cluster were diverse. Of the seven groups, group 1, 5 and 12 belonged to Zn-CDFs, group 6 and 7 were constructed Zn/Fe-CDFs, and group 8 and 9 made up Mn-CDFs. Furthermore, group 1 contained 14 MTP proteins, which were AtMTP1, AtMTP2, AtMTP3, AtMTP4, OsMTP1, SbMTP1, CsMTP1, CsMTP3, CsMTP4, VvMTP1, VvMTP4, FtMTP1, FtMTP3.1 and FtMTP3.2. Group 5 was composed of 6 MTP proteins, i.e., AtMTP5, OsMTP5, SbMTP5, CsMTP5, VvMTP5 and FtMTP5. Similarly, group 6 was composed of six MTP proteins, AtMTP6, OsMTP6, SbMTP6, CsMTP6, VvMTP6 and FtMTP6. Besides, seven MTP proteins, AtMTP7, OsMTP7, SbMTP7, CsMTP7, VvMTP7, FtMTP7.1 and FtMTP7.2 formed group 7. For group 8, there were 11 MTP proteins, namely AtMTP8, OsMTP8, OsMTP8.1, SbMTP8, CsMTP8.1, CsMTP8.2, VvMTP8, FtMTP8.1 and FtMTP8.2. The members of group 9 were seventeen, with AtMTP9, AtMTP10, AtMTP11, OsMTP9, OsMTP11, OsMTP11.1, SbMTP9, SbMTP11, CsMTP9, CsMTP10, CsMTP11.1, CsMTP11.2, VvMTP9, VvMTP9.1, VvMTP11, FtMTP9 and FtMTP11. Meanwhile, six MTP proteins including AtMTP12, OsMTP12, SbMTP12, CsMTP12, VvMTP12 and FtMTP12 belonged to group 12.

### 2.3. Phylogenetic Tree, Gene Structure and Motif Patterns of FtMTPs

**Figure 2:**
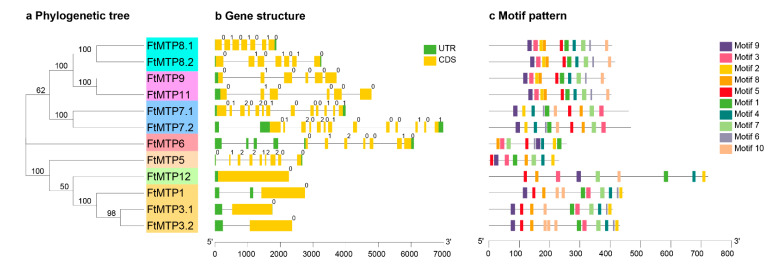
Phylogenetic tree, gene structure, and motif pattern analysis of *FtMTPs*. (**a**) the neighbor-joining phylogenetic tree was constructed with MEGA 7.0 using FtMTP amino acid sequences with 1000 replications bootstrap; (**b**) exon-intron structure of *FtMTP* genes where yellow boxes represented the exons, the black lines represented the introns, and the green boxes represented the untranslated regions (UTRs), with size scales detailed at the bottom; (**c**) the motif patterns of FtMTP proteins using ten conserved motifs (Supplementary Figure S1) are represented by the unique color mentioned in the legend on the top lift, with size scales detailed at the bottom.

As shown in Figure 2a, Tartary buckwheat MTP gene family members were divided into three clusters (Zn-CDFs, Zn/Fe-CDFs, and Mn-CDFs) and seven groups (1, 5, 6, 7, 8, 9, and 12). Of the seven groups, groups 1, 5, and 12 belonged to Zn-CDFs, groups 6 and 7 were constructed Zn/Fe-CDFs, and groups 8 and 9 made up Mn-CDFs. Group 1 was the largest with three members, followed by groups 7, 8, and 9 (2 genes), whereas groups 5, 6, and 12 each had only one gene. All *FtMTPs* contained exon and intron; however, the number and length of exon or intron were diverse among different clusters (Figure 2b). Regarding exons, Zn/Fe-CDFs had 7 or 13 exons, Mn-CDFs contained 6 or 7 exons, and Zn-CDFs had only one exon expect for group 5 (9 exons). For introns, Zn/Fe-CDFs were the largest with 12–15 introns, followed by Mn-CDFs (6–8 introns), whereas Zn-CDFs had 1–2 introns except for Group 5 (10 introns). The conserved motifs of FtMTP proteins were predicted and analyzed using MEME depending on the amino acid sequences with ten motifs (Figure 2c). It was primarily observed that the number, type, or order of motifs was similar within the same group in addition to that among different clusters. These ten motifs appeared at least once in groups 1, 7, 8, and 9, whereas groups 6 and 12 lacked motifs 10 and 6, respectively, and Group 5 lost both motifs 6 and 10. In addition, FtMTP1 and FtMTP3.2, two members belonged to Group 1, containing two and three motif 10, respectively.

### 2.4. Structure Variation of the FtMTP Proteins

**Figure 3:**
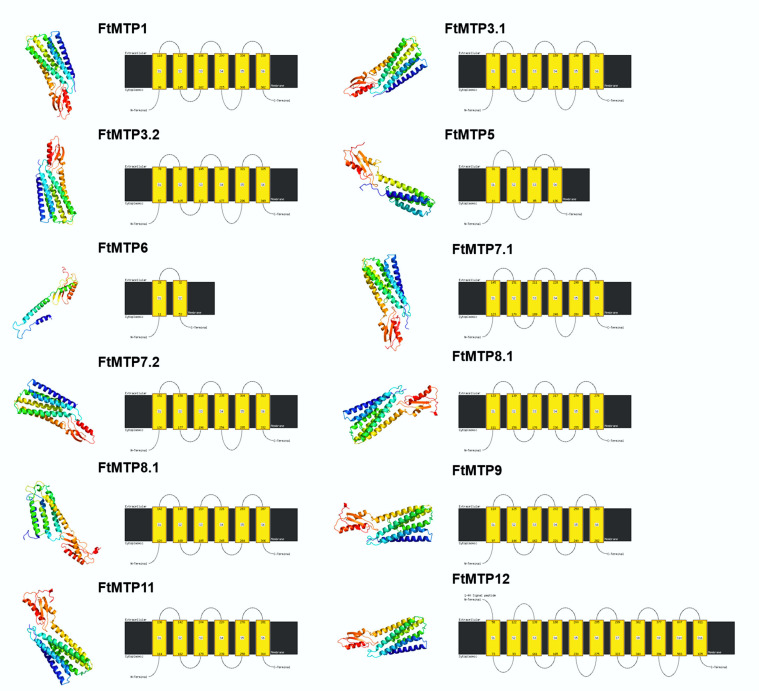
Structure prediction analyses of FtMTP proteins. Models had been generated by using Phyre 2 server in normal mode and were visualized by rainbow color from N to C terminus and organized in FtMTP1, and FtMTP3.1 to FtMTP12. For the left pictures, the coils and the smooths represented alpha-helices and beta-sheets, respectively; For the right pictures, the black line represented protein sequences, and the yellow boxes represented transmembrane helices.

To further explore the structure variation, twelve FtMTP proteins were modeled using Phyre2 with normal mode, and the results were shown in Figure 3. It could be clearly seen that all FtMTP proteins contained α-helices and β-sheets. Expect for FtMTP7.2, which contained two β-sheets, the rest of the proteins contained three β-sheets, and except for FtMTP5 and FtMTP6, which contain six and four α-helices, respectively, the rest of the proteins contained eight α-helices. Besides that, three members, FtMTP1, FtMTP3.1, and FtMTP3.2, in Group 1 contained six transmembrane helices. Proteins belonging to groups 5 and 7 had four transmembrane helices. There was only two transmembrane helices in Group 6, which was the least of all seven groups. Group 8 had four (FtMTP8.2) or five (FtMTP8.1) transmembrane helices, whereas Group 9 had four (FtMTP11) or six (FtMTP9) transmembrane helices, and Group 12 contained the largest number (10) of transmembrane helices among seven groups. Based on these results, the numbers of transmembrane helices of the FtMTP proteins in the same group were similar, whereas variable among different groups, indicating that the diversity of the structure endowed the functional complexity of the *FtMTP* gene family.

### 2.5. Cis-Regulatory Elements of the FtMTPs

**Figure 4:**
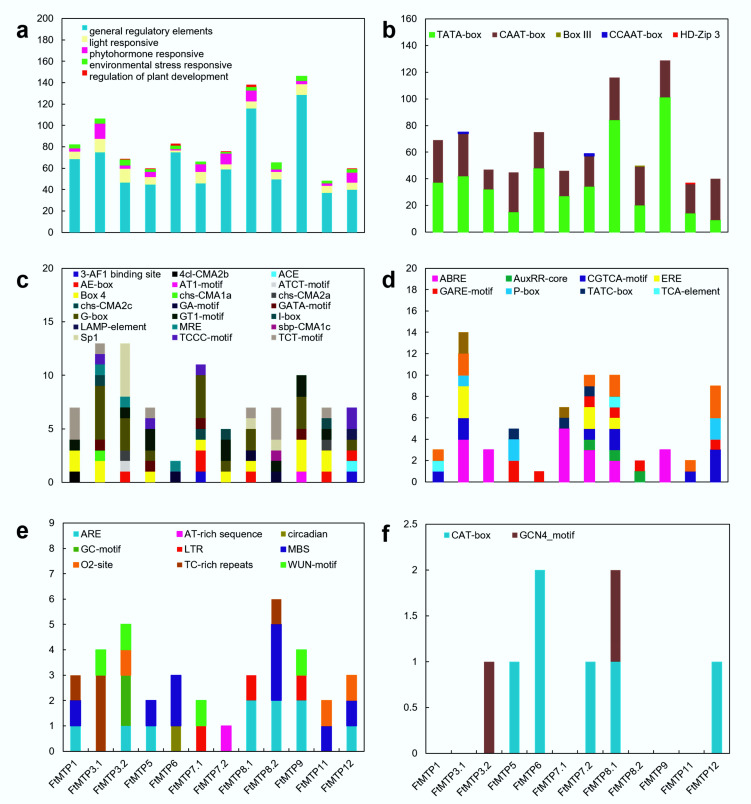
*Cis*-regulatory elements (*CREs*) of *FtMTP* genes. (**a**) members and numbers of *CREs* of *FtMTP* genes; (**b**) members and numbers of general regulatory elements of *FtMTP* genes; (**c**) members and numbers of light-responsive elements of *FtMTP* genes; (**d**) members and numbers of phytohormone responsive elements of *FtMTP* genes; (**e**) members and numbers of environmental stress-responsive elements of *FtMTP* genes; (**f**) members and numbers of regulation of plant development elements of *FtMTP* genes. The value used in the vertical axis in these figures represented the number of corresponding *cis*-regulatory elements.

To better understand the transcription regulatory mechanism of *FtMTP* genes, *cis*-regulatory elements (*CREs*) in the 1500 bp upstream of these genes were analyzed. After removing unknown motifs, a total of 47 *CREs* were found in the promoter of *FtMTP* genes, and these *CREs* were further classified into five groups, general regulatory elements, light response elements, hormone response elements, environmental stress response elements, and regulation of plant development elements (Figure 4a). The overall frequency of these *CREs* in *FtMTP* genes and their frequency in the upstream region of each corresponding gene were very diverse. General regulatory elements, light response elements, hormone response elements, and environmental stress response elements were frequently found in all of the *FtMTP* genes, but regulation of plant development elements was specifically found in *FtMTP3.2*, *FtMTP5*, *FtMTP6*, *FtMTP7.2*, *FtMTP8.1*, and *FtMTP12*. Further analysis showed that five types of general regulatory elements, including TATA-box, CAAT-box, Box Ⅲ, CCAAT-box, and HD-ZIP 3, were identified in the upstream region of *FtMTP* genes. TATA- and CAAT-boxes were frequently found in all of the *FtMTP* genes, while Box Ⅲ was specifically found in *FtMTP8.2*, CCAAT-box was specifically present in *FtMTP3.1*, and HD-ZIP 3 specifically existed in *FtMTP11* (Figure 4b). There were 21 types of light-responsive elements and ten types of hormone response elements involved in ethylene, abscisic acid, auxin, MeJA, salicylic acid, and gibberellin mediated responses were observed in all of the *FtMTP* genes (Figure 4c,d). These hormone response elements identified in *FtMTP8.1* might participate in these six plant hormones mediated responses. However, only ABRE or GARE-motif involved in abscisic acid or gibberellin-mediated responses were resolved in *FtMTP3.2* and *FtMTP6*, while *FtMTP5* contained ABRE, GARE-motif, and TATC-box elements. Nine types of environmental stress response elements involved in anaerobic induction, stress and defense, drought, low temperature, and other abiotic stresses were frequently found in all of the *FtMTP* genes (Figure 4e). Most *FtMTP* genes shared ARE element, which was essential for the anaerobic stress responses, while MBS element (MYB binding site), which was involved in drought stress responses, were specifically found in *FtMTP5*, *FtMTP6*, *FtMTP8.2*, *FtMTP11* and *FtMTP12*, and LTR element involved in low-temperature stress were specifically found in *FtMTP7.1*, *FtMTP8.1*, and *FtMTP9*. *CREs* involved in the regulation of plant development elements, i.e., CAT-box and GCN4_motif, which were essential for meristem or endosperm development, were also investigated (Figure 4f). It could be clearly found that GCN4_motif was specifically found in *FtMTP3.2* and *FtMTP8.1*, yet CAT-box was specifically observed in *FtMTP5*, *FtMTP6*, *FtMTP7.2*, *FtMTP8.1*, and *FtMTP12*.

### 2.6. Tissues-Specific Expression Profile of FtMTPs

**Figure 5:**
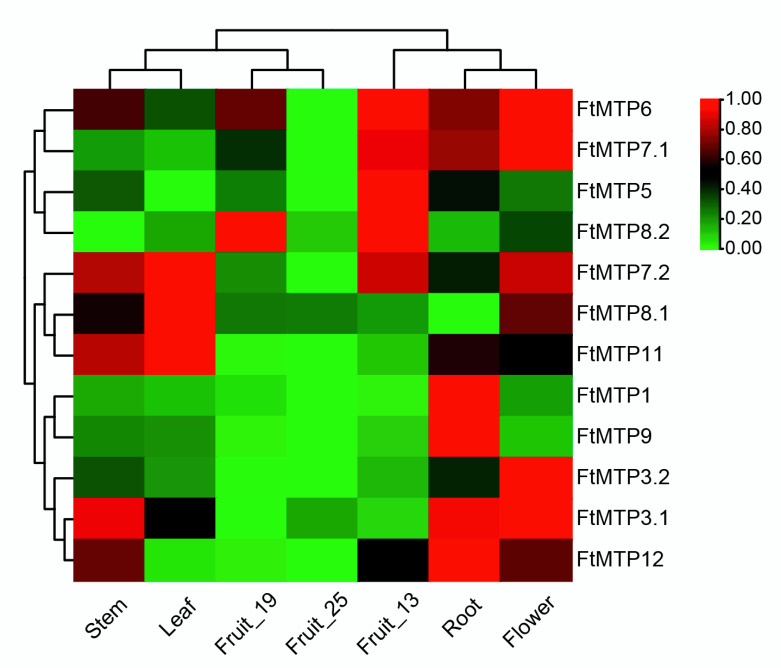
Heatmap analysis of the expressions of *FtMTP*s in different Tartary buckwheat tissues at different developmental stages. Gene expression (FPKM) was normalized and visualized using the TBtools software. The Stem, Leaf, Fruit_19, Fruit_25, Fruit_13, Root, and Flower marked in the figure represented the stem, leaf, fruit at 19 days of development, fruit at 25 days of development, fruit at 13 days of development, root and flower of Tartary buckwheat, respectively.

In Figure 5, it showed the expression of *FtMTP*s from different Tartary buckwheat tissues under different developmental stages, including root, stem, leaf, flower, fruit at 13 days of development (fruit_13), fruit at 19 days of development (fruit_19), fruit at 25 days of development (fruit_25). From the results, *FtMTP3.1* and *FtMTP3.2*, which belonged to Group 1, expressed highest in flower, and lowest in fruit_19 or fruit_25, but the other Group 1 member, *FtMTP1*, had the highest and the lowest expression in root and fruit_13, respectively. A member of Group 5, *FtMTP5*, showed the highest and lowest expression levels in fruit_13 and leaf, respectively. *FtMTP6*, the gene from Group 6 displayed the highest expression in flower and fruit_13, and the lower expression in fruit_25. In addition, all members in groups 7 and 8 showed tissue-specific expression patterns. *FtMTP7.1* was expressed highly in flowers, while *FtMTP7.2* was strongly expressed in leaves. The expression of *FtMTP8.1* in leaves was highest compared to other tissues; however, *FtMTP8.2*, the other member in Group 8, showed the highest expression in both fruit_13 and fruit_19. *FtMTP9* was strongly expressed in roots and weakly expressed in fruit_19 and fruit_25, and *FtMTP11* exhibited the highest and lowest expression in leaves and fruit_25, respectively. *FtMTP12*, a Group 12 member, expressed higher in roots, stems, flowers, and fruit_13, but lower in leaf, fruit_19 and fruit_25.

### 2.7. Expression of FtMTPs under Multiple Metal Stress

**Figure 6:**
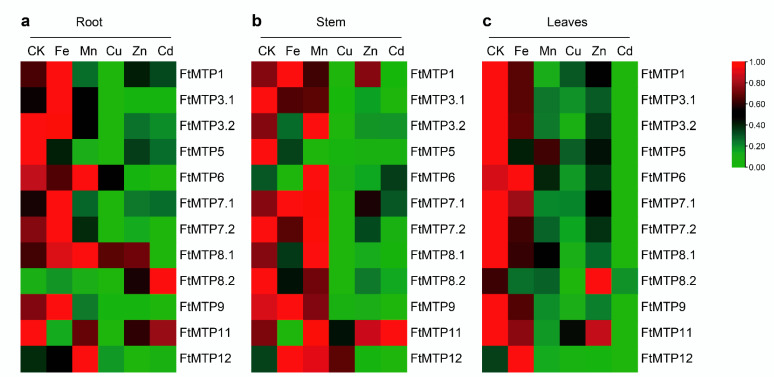
Heatmap expressions of the abundance of *FtMTPs* in different Tartary buckwheat tissues under multiple metal stress. (**a**) heatmap analysis of the abundance of *FtMTPs* in Tartary buckwheat root, gene expression was normalized using zero to one method; (**b**) heatmap analysis of the abundance of *FtMTPs* in Tartary buckwheat stem, gene expression was normalized using zero to one method; (**c**) heatmap analysis of the abundance of *FtMTPs* in Tartary buckwheat leaves. Gene expression was normalized and visualized by the TBtools software. CK, Fe, Mn, Cu, and Zn, appearing in Figure 6a–c, represent the root, stem, and leaves of Tartary buckwheat under normal conditions, Fe^3+^, Mn^2+^, Cu^2+^, and Zn^2+^ treatments, respectively.

The expression of *FtMTP*s in Tartary buckwheat roots under normal conditions and five metal stresses were investigated by qRT-PCR and are shown in Figure 6a. Compared with the expression of *FtMTP*s in roots under normal condition (CK), Fe^3+^ treatment upregulated the expression of *FtMTP1*, *FtMTP3.1*, *FtMTP7.1*, *FtMTP8.1*, and *FtMTP8.2*, whereas downregulated the expression levels of *FtMTP5* and *FtMTP11*. Similarly, Mn^2+^ treatment upregulated the expression of *FtMTP8.1* and *FtMTP12*, while downregulated the expression of *FtMTP1*, *FtMTP3.1*, *FtMTP5*, *FtMTP7.1*, *FtMTP7.2*, and *FtMTP9*. Except for *FtMTP8.1*, the expression of all *FtMTP*s was reduced under Cu^2+^ treatment. Zn^2+^ treatment resulted in the expression of *FtMTP8.2* upregulation, while the expression of *FtMTP1*, *FtMTP3.1*, *FtMTP3.2*, *FtMTP5*, *FtMTP6*, *FtMTP7.1*, *FtMTP7.2*, *FtMTP9*, and *FtMTP12* downregulation. Only the expression of *FtMTP8.2* was increased in response to Cd^2+^ treatment, and the expression levels of the remaining *FtMTP*s except for *FtMTP11* were reduced.

The expression of *FtMTP*s in Tartary buckwheat stems under normal condition and five metal stresses were examined and showed in Figure 6b. Compared with the expression of *FtMTP*s in Tartary buckwheat stems under normal condition (CK), Fe^3+^ treatment induced the expression of *FtMTP12*, whereas reduced the expression of *FtMTP3.1*, *FtMTP3.2*, *FtMTP5*, *FtMTP6*, *FtMTP7.2*, *FtMTP8.1*, *FtMTP8.2*, and *FtMTP11*. Similarly, Mn^2+^ treatment resulted in the expression of *FtMTP3.2*, *FtMTP6*, *FtMTP8.1*, and *FtMTP12* upregulation, while the expression of *FtMTP3.1*, *FtMTP5*, *FtMTP8.2*, and *FtMTP9* downregulation. Moreover, the expression of *FtMTP6*, *FtMTP8.1*, and *FtMTP12* were upregulated in response to Cu^2+^ treatment, but the expression of *FtMTP1*, *FtMTP3.1*, *FtMTP3.2*, *FtMTP5*, *FtMTP8.2*, and *FtMTP9* were downregulated. Expect for *FtMTP1*, *FtMTP6*, *FtMTP7.1*, and *FtMTP11*, the expression of the remaining eight *FtMTP*s were reduced under Zn^2+^ treatment. Besides, the expression of *FtMTP1*, *FtMTP3.1*, *FtMTP3.2*, *FtMTP5*, *FtMTP7.1*, *FtMTP7.2*, *FtMTP8.1*, *FtMTP8.2*, *FtMTP9*, and *FtMTP12* were downregulated under Cd^2+^ treatment.

The expression of *FtMTP*s in Tartary buckwheat leaves under normal condition and five metal stresses were tested by qRT-PCR. As shown in Figure 6c, when compared to the expression of *FtMTP*s in Tartary buckwheat leaves under normal condition (CK), the expression of *FtMTP12* upregulated in response to Fe^3+^ treatment, whereas the expression of most *FtMTP*s, including *FtMTP1*, *FtMTP3.1*, *FtMTP3.2*, *FtMTP5*, *FtMTP7.2*, *FtMTP8.1*, *FtMTP8.2*, and *FtMTP9* downregulated. Mn^2+^, Cu^2+^, and Cd^2+^ treatments resulted in the expression of all *FtMTP*s reduced. Besides, except that the expression of *FtMTP8.2* was upregulated under Zn^2+^ treatment, the expression of the remaining *FtMTP*s under Zn^2+^ treatment was reduced.

## 3. Discussion

Soil heavy metal pollution caused by artificial activities such as mineral smelting, sewage irrigation, and large-scale application of pesticides and fertilizers has become one of the abiotic factors restricting crop growth and production, thus food safety [5,6]. Metal tolerance proteins (MTPs) as divalent cation transporters involve in metal uptake and transportation and play vital roles in metal tolerance and detoxification of crops. In the present study, a total of 12 *FtMTPs* were identified in *F.* *tartaricum*, which is similar to that in *A.* *thaliana* [15], but higher than that in *O. sativa* [15], *V. vinifera* [26], and *S. bicolor* [25] and less than that in *C. sinensis* [27], *P. trichocarpa* [35], and *N. tabacum* [36]. However, homologs of *AtMTP2*, *AtMTP4*, and *AtMTP10* could not be detected in the *F. tartaricum*, whereas two *AtMTP3* orthologs, *FtMTP3.1* and *FtMTP3.2*, two *AtMTP7* orthologs, *FtMTP7.1* and *FtMTP7.2*, and two *AtMTP8*, *FtMTP8.1* and *FtMTP8.2*, were identified (Table 1 and Figure 1). These results indicate that gene loss and duplication might have occurred in the evolution of the MTP gene family in *F.* *tartaricum*. Meanwhile, the characteristic analysis of FtMTP proteins was processed, and all FtMTP proteins were predicted to be located in the vacuolar membrane (Table 1), suggesting that FtMTPs might mainly participate in the transportation of heavy metals to vacuoles. Interestingly, the *FtMTP9* was also predicted to be located in the cell membrane. This result indicated that *FtMTP9* might play an essential role in heavy metal transportation between Tartary buckwheat cells. The function of *FtMTP9* for heavy metal tolerance in Tartary buckwheat needs to be investigated in future studies.

There were three structural features of MTP proteins, an N-terminal signature sequence, a cation efflux domain, and approximately six predicted transmembrane regions [13]. In this study, the cation efflux domain and the modified signature sequence were found in all FtMTP proteins, although some other motifs were not present or more than one in certain FtMTP members, as shown in Figure 2. Besides, FtMTP6 had the smallest peptide length (256 amino acid), MW (28.54 KDa), and TMHs (1 TMHs), while FtMTP12 had the largest peptide length (722 amino acid), MW (80.51 KDa), and TMHs (10 TMHs) (Table 1 and Figure 3). These results were consistent with the characteristics of the MTP gene family in other plant species, including *C. sinensis* [27], *P. trichocarpa* [35], and *N. tabacum* [36], which indicated that the structure and function conservation of the MTP gene family in plant evolutionary events.

In general, phylogenetic analysis is one of the strategies to predict the function of uncharacterized genes [37]. For instance, the reported gene families in *F. tartaricum*, including NAC [38], AP2/ERF [39], GRAS [40], ARF [41], bZIP [42], Bhlh [43], MADS [44], SPL [45], BBX [46], andNF-Y [47], showed similar gene functions between the phylogenetic orthologs. This study showed FtMTPs and AtMTPs were extremely similar at the protein level, and the results of the phylogenetic analysis showed that their orthologs were closely clustered together (Figure 1), thus indicating *FtMTP*s might have similar functions with their corresponding *AtMTP* orthologs.

Gene expression regulation is involved in different stages of plant growth and development. Normally, transcriptional regulation and post-transcriptional regulation are two aspects in which gene expression regulation happens [35]. Transcriptional regulation is an important way to regulate gene expression level, while cis-acting regulatory elements involving in this process through interactions with RNA polymerase and specific transcription factors [35]. Here, different cis-acting regulatory elements were processed in the upstream region of each *FtMTP* (Figure 4), which revealed regulating events at the initiation of transcription. All *FtMTP* genes contained light-responsive cis-acting elements, phytohormone responsive cis-acting elements, environmental stress-response cis-acting elements, and also general regulatory elements, as shown in Figure 4, indicating that the expression of *FtMTPs* genes could be affected by photoperiod, plant hormones, and environmental stress. This result also implied that all MTPs genes of Tartary buckwheat could be transcriptionally regulated by multiple stimulates. Additionally, only *FtMTP3.2*, *FtMTP5*, *FtMTP6*, *FtMTP7.2*, *FtMTP8.1*, and *FtMTP12* contained cis-acting elements related to plant development regulation, indicating that these *FtMTPs* might also be related to the growth and development of Tartary buckwheat. In particular, *FtMTP9* was detected to have the largest number of regularity elements, suggesting that it might be involved in many regulation pathways. Overall, abundant cis-acting regulatory elements located upstream of these *FtMTPs* imply that they might play an important role not only in heavy metal stress responses but also in multiple stress responses and plant development.

In addition, analysis of gene expression patterns could also provide insights into gene functions [37]. Comprehensive analysis of expression patterns of *FtMTP*s in three tissues under five metal stresses (Figure 5 and Figure 6), this study formed a relatively comprehensive function prediction of *FtMTP*s. Interestingly, previous studies reported that Zn-CDFs such as *AtMTP1*, *AtMTP3*, *AtMTP5*, *AtMTP12*, and their orthologs, *PtdMTP1*, *CsMTP1*, *CitMTP1*, *CisMTP3*, *CitMTP5*, and *CitMTP12* were mainly involved in Zn and Cd detoxification [17,20,37,48,49]; however, in this study, only the member of Zn-CDFs, *FtMTP1* were induced in the stems after treatment with excessive amounts of Zn, and no Zn-CDFs in *F. tartaricum* were induced in the roots, stems or leaves after treatment with excessive amounts of Cd. This may be attributed to the Zn and Cd concentrations used in our experiment, which means that the response of these genes may require appropriate concentrations of Zn and Cd treatment. Besides, this study also found that the Zn-CDF in *F. tartaricum* except for *FtMTP5* induced in the roots, stem, or leaves after exposure to high levels of Fe, suggesting the potential roles of these genes in Fe homeostasis, which are consistent with previous research. Mn-CDF, such as the members of *MTP8*, *MTP9*, *MTP10*, and *MTP11*, play vital roles in Mn transport and detoxification [22,24,50,51,52]. In the present study, *FtMTP8.1* in roots and stems were highly expressed after the Mn treatment; in addition, the expression of *FtMTP8.1* in roots was also induced by Fe or Zn treatment. For the other three Mn-CDFs in *F. tartaricum*, the expression of *FtMTP8.2* in roots and leaves were upregulated by Cd and Zn treatment, respectively, *FtMTP9* in roots and stems were induced after Fe treatment, and *FtMTP11* in stems was upregulated by Mn, Zn, and Cd treatment. This finding supports those of previous studies. For Zn/Fe-CDFs, including *MTP6* and *MTP7*, it is mainly involved in the uptake, transport, and homeostasis of Fe or Zn. This study found that *FtMTP7.1* and *FtMTP7.2* in roots, *FtMTP7.1* in stems were highly expressed after excessive Fe treatment, and FtMTP6 in roots and stems and *FtMTP7.1* in stems were upregulated by Mn treatment, which has been proven by previous studies. Although most of the MTP genes have been fully characterized in many plant species, the role of MTP genes in Cu tolerance and detoxification in plants remains unknown. From the result of this study, the expression of *FtMTP8.1* in roots and the expression of *FtMTP12* in stems were significantly upregulated after exposure to excessive amounts of Cu. Similar results have been found in *C. sinensis* [37]. Therefore, more in-depth investigations on Cu detoxification of these *MTP* genes need to be carried out in the future.

## 4. Materials and Methods

### 4.1. Identification, Physicochemical Parameters of MTPs in Tartary Buckwheat

Tartary buckwheat genome was downloaded from MBKBASE (http://mbkbase.org (accessed on 20 October 2021)). Protein sequences of 55 *MTPs* in *Arabidopsis thaliana*, *Oryza sativa*, *Sorghum bicolor*, *Vitis vinifera* and *Camellia sinensis* (Supplementary Table S1) were downloaded from the National Center for Biotechnology (NCBI) (https://www.ncbi.nlm.nih.gov/ (accessed on 20 October 2021)), the Rice Genome Annotation Project [53] (http://rice.uga.edu/ (accessed on 20 October 2021)) and Phytozome [54] (https://phytozome-next.jgi.doe.gov/ (accessed on 20 October 2021)) respectively. In order to identify the candidate *MTP* genes in Tartary buckwheat genome, all known *Arabidopsis MTP* genes were used as query sequences to search against the Tartary buckwheat genome by a BLASTp search at a score value of ≥ 100 and e-value ≤ 1 × 10^−5^ [55]. After that, the *MTP* gene sequences were retrieved from the Tartary buckwheat genome. Then, domains and functional sites in each Tartary buckwheat candidate *MTP* gene were examined with Pfam tool [56] (http://pfam.xfam.org/ (accessed on 23 October 2021)) and Smart program [57] (http://smart.embl.de/smart/batch.pl (accessed on 23 October 2021)), and all candidate Tartary buckwheat *MTP* genes containing cation efflux family (CDF) domain (PF01545) were verified. After removing redundant sequences, 12 *MTP* genes (Supplementary Table S2) in the Tartary buckwheat genome were successfully identified. Subsequently, the ProtParam tool of ExPaSy [58] (https://www.expasy.org/ (accessed on 5 November 2021)) was used to calculate the molecular weight (MW), the theoretical isoelectric point (pI), and the grand average of hydropathicity (GRAVY) of proteins. Putative transmembrane regions in proteins were predicted using the TMHMM Server V2.0 [59] (http://www.cbs.dtu.dk/services/TMHMM/ (accessed on 5 November 2021)). Subcellular localization (SL) of FtMTP proteins were predicting with the Plant-mPLoc version 2.0 [60,61,62,63] (http://www.csbio.sjtu.edu.cn/bioinf/plant-multi/ (accessed on 5 November 2021)).

### 4.2. Phylogenetic Analysis

ClustalW [64] was used for *MTP* gene sequences alignment among *Fagopyrum tartaricum*, *Arabidopsis* *thaliana*, *Oryza sativa*, *Sorghum bicolor*, *Vitis vinifera*, and *Camellia sinensis.* Phylogenetic trees were constructed using MEGA 7.0 software [65] with neighbor-joining method [66] and 1000 bootstrap test replicates [67], then drawn with EvolView V3 [68] (https://www.evolgenius.info/evolview/ (accessed on 10 December 2021)).

### 4.3. Gene Structure and Motif Analysis

Intron-exon structures of *FtMTP* genes were analyzed using the TBtools software [69]. Pfam tool [56] (http://pfam.xfam.org/ (accessed on 12 December 2021)) and MEME program [70] (https://meme-suite.org/meme/ (accessed on 12 December 2021)) were used to analyze conserved domains and motifs in *FtMTP* gene sequences, respectively. Subsequently, domain and motif diagrams were drawn with the TBtools [69].

### 4.4. Protein Modeling and Prediction

All FtMTP amino acid sequences were submitted to Phyre2 (http://www.sbg.bio.ic.ac.uk/~phyre2/ (accessed on 20 December 2021)) and 3D protein structure models for *FtMTP* genes were Predicted with homology modeling under the normal mode [71].

### 4.5. Cis-Regulatory Elements Prediction

The promoter sequence for each *FtMTP* gene was defined as 1500 bp upstream from the start codon and extracted with TBtools software [69]. All the *cis*-regulatory elements (*CREs*) in promoter sequence were analyzed and identified by the PlantCARE database [72] (http://bioinformatics.psb.ugent.be/webtools/plantcare/html/ (accessed on 30 December 2021)).

### 4.6. Transcriptome Data Analysis

The expression data of Tartary buckwheat MTP genes in seven tissues, including root, stem, leaf, flower, and fruit under three development stages, were obtained from the Tartary buckwheat Database [73] (http://tbd.sicau.edu.cn/index/gev/index.html (accessed on 10 January 2022)), and the HeatMap Illustrator of TBtools software [69], were used for visualization of the expression data.

### 4.7. Plant Cultivation and Stress Treatments

Tartary buckwheat seeds were sown in soil pots under natural conditions. After growth for one week, buckwheat seedlings were transplanted into uniform pots (d = 9.4 cm, h = 8 cm) with uniform soil (one seedling in a pot) under greenhouse conditions (22 to 25 °C, 12 h light and 12 h darkness, 50% to 60% relative humidity). Three days later, plants were irrigated with five metal ion solutions respectively. Sources and concentrations of metal ions are shown in Table 2. Plants were irrigated with 100 mL water (controls) or solution (treatments), and the pots were placed in culture dishes. After treatment for three days, roots, stems, and leaves from the same plant of each treatment were harvested separately, washed with water three times, frozen by liquid nitrogen, ground to powder, and then stored at −80 °C for RNA isolation. Three biological replicates were used for each treatment.

### 4.8. RNA Extraction and Quantitative Real-Time PCR (qRT-PCR) Analysis

Total RNAs were isolated using the Eastep™ Super Total RNA Extraction Kit (Promega, Madison, WI, USA) according to the manufacturer’s instructions. The purity, integrity, and concentration of RNA were determined with a NanoDrop1000 (NanoDrop Technologies, Inc., Wilmington, DE, USA). Furthermore, 3 µg of RNA was reverse-transcribed to cDNA using a GoScript ^TM^ Reverse Transcription System (Promega, Madison, WI, USA). The optimal forward and reverse primers (Supplementary Table S3) designed by an online tool Primer-BLAST10 was used for qRT-PCR analysis. Then, qRT-PCR was conducted in triplicate using cDNAs prepared from different tissues with various treatments with a FastStart Universal SYBR Green Master (Rox, Roche, Indianapolis, IN, USA) and the ABI 7500 Sequence Detection System (Applied Biosystems, Foster City, CA, USA). Thermal cycling parameters were as follows: 95 °C for 10 min, followed by 40 cycles of 94 °C for 5 s, 60 °C for 15 s, and 72 °C for 34 s. *FtH_3_* gene was amplified as an internal control. Relative gene expression levels were obtained by dividing extrapolated transcript levels of target genes by levels of *FtH_3_* (the internal control) from the same sample.

### 4.9. Statistical Analysis

All statistical analyses were performed using SPSS version 18.0 (SPSS Inc., Chicago, IL, USA), and results were expressed as means ± SD. The gene expression was normalized using the TBtools software.

## 5. Conclusions

In this study, 12 MTP protein-encoding gene members were identified from the Tartary buckwheat genome, and they were clustered into seven groups based on their phylogenetic relationships. The physicochemical parameters and three-dimensional structures of proteins belonging to the same group were similar but varied among different groups. The analysis of cis-acting elements showed that the expression of the *FtMTP* gene family might be regulated by various environmental signals such as plant growth, development, light response, and stress responses. Besides, the expression of the *FtMTP* gene family was specific to different tissues or different developmental stages of plants, and the expression of most *FtMTP* genes could be induced by specific metal ions. These findings will provide basic and useful information for future research on the metal tolerance mechanisms and the genetic improvement of *Fagopyrum tataricum*.

## Figures and Tables

**Table 1:** Sequences and characteristic analysis of the FtMTP proteins.

Gene Names	Peptide Length	pI ^a^	MW ^b^ (KDa)	TMHs ^c^	GRAVY ^d^	SL ^e^
FtMTP1	445	5.98	49.58	6	−0.041	V ^f^
FtMTP3.1	408	6.32	44.74	6	0.050	V
FtMTP3.2	431	6.19	46.95	6	−0.016	V
FtMTP5	230	6.03	25.41	4	0.374	V
FtMTP6	256	5.9	28.54	1	0.009	V
FtMTP7.1	460	9.23	51.39	4	0.002	V
FtMTP7.2	467	6.85	51.10	4	0.066	V
FtMTP8.1	405	5.11	45.61	5	−0.065	V
FtMTP8.2	415	5.57	46.58	4	0.016	V
FtMTP9	384	6.88	43.78	6	−0.018	C ^g^/V
FtMTP11	403	5.08	45.40	4	0.037	V
FtMTP12	722	6.62	80.51	10	0.032	V

^a^ pI: predicted isoelectric point; ^b^ MW: predicted molecular weight; ^c^ TMHs: predicted transmembrane helices; ^d^ GRAVY: grand average of hydropathicity; ^e^ SL: subcellular localization; ^f^ V: vacuole; ^g^ C: cell membrane.

**Table 2:** Concentrations and sources of different metal ions used in this study.

Metal Ion	Concentration (mg L^−1^)	Source
Zn	2.0	ZnSO_4_·7H_2_O
Cu	5.0	CuSO_4_·5H_2_O
Mn	5.0	MnSO_4_
Fe	2.5	FeCl_3_·6H_2_O
Cd	2.0	CdCl_2_·2.5H_2_O

## Data Availability

Data is contained within the article or Supplementary Materials.

## Supplementary Materials

The following supporting information can be downloaded at: https://www.mdpi.com/article/10.3390/plants11070850/s1, Figure S1: The detailed information about ten motifs of FtMTP proteins. Table S1: Protein sequences of 55 MTPs used in this study, Table S2: Sequences of FtMTPs, Table S3: Optimal forward and reverse primers for qRT-PCR analysis.
