# Supplementary figures and images for: Metal Tolerance Protein Encoding Gene Family in Fagopyrum tartaricum: Genome-Wide Identification, Characterization and Expression under Multiple Metal Stresses
Source: Plants (Basel). 2022 Mar 23;11(7):850. doi: 10.3390/plants11070850 (PMC9003181; doi:10.3390/plants11070850)

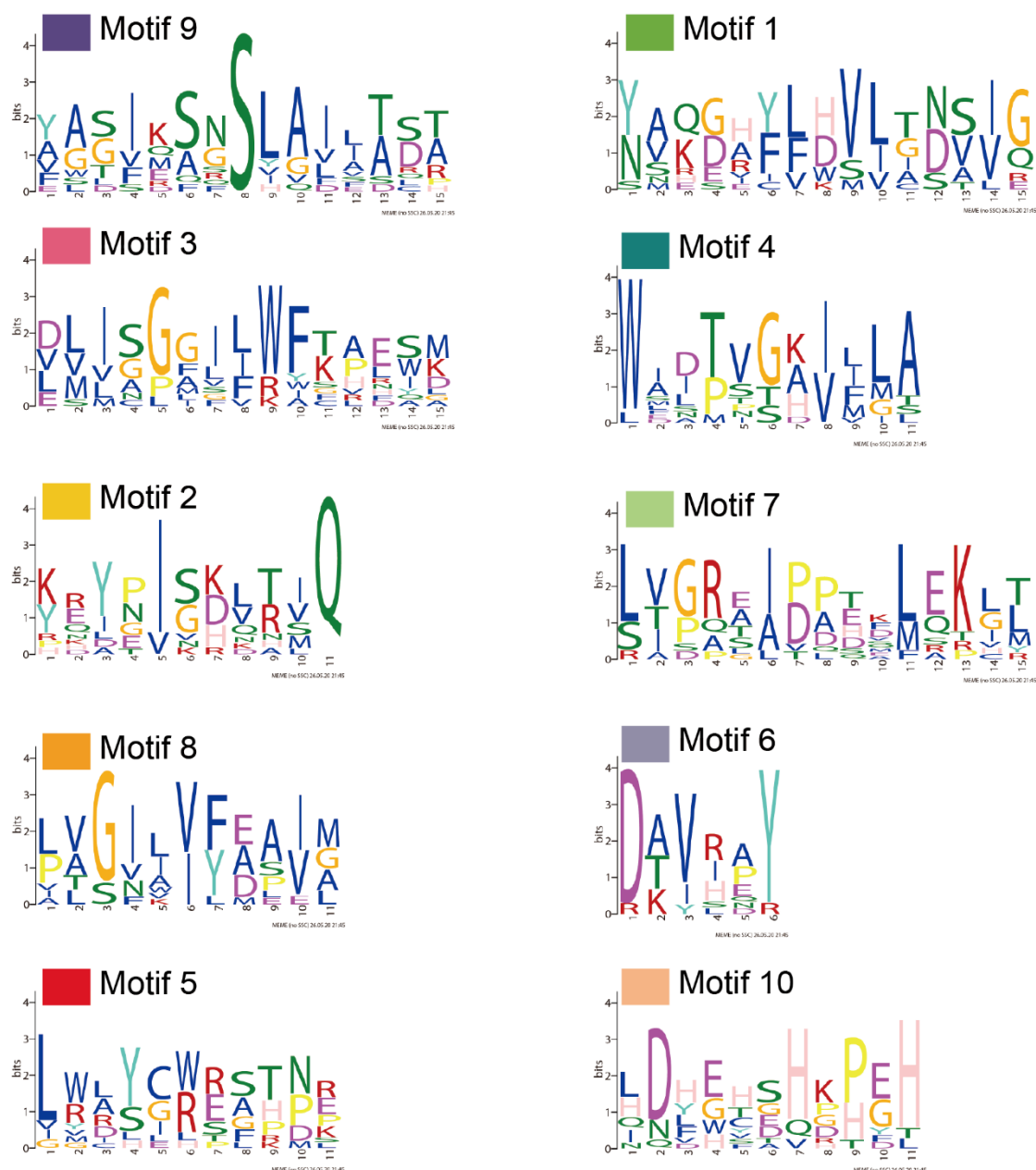

**Figure S1.** The detailed information about ten motifs of FtMTP proteins.

Supplement: Supplementary file 1 [file plants-11-00850-s001.zip › Supplementary Figure S1 .pdf]
